# Validation of the Human Progesterone Assay Kit for Cattle as a Pregnancy Diagnosis Tool

**DOI:** 10.1155/2022/4610830

**Published:** 2022-04-21

**Authors:** Tesfaye Engida, Fikire Lobago, Alemayehu Lemma, Anteneh M. Yenehun, Berhane Mekete

**Affiliations:** ^1^Ambo University, College of Agriculture and Veterinary Science, Department of Animal Sciences, Ambo, Ethiopia; ^2^Addis Ababa University, College of Veterinary Medicine and Agriculture, Bishoftu, Ethiopia; ^3^University of Nebraska, East Campus, Department of Animal Sciences and Veterinary Medicine, Nebraska, Lincoln, USA

## Abstract

Accurate pregnancy diagnosis is an important criterion and management tool for successful dairying. Early identification of non-pregnant dairy heifers and cows after breeding can improve pregnancy rate and life time production. Determination of progesterone hormone levels is more accurate to diagnose failed pregnancies in dairy animals. This method is not always available in developing countries. Some of the kits available are developed for humans and might be used for cattle because in principle, progesterone is not species-specific and detection methods are the same in both animals and human beings. The study aimed at validating a human progesterone ELISA kit for use in cattle as a pregnancy diagnosis tool. Forty Boran and crossbred cattle (22 pregnant and 18 non-pregnant) were selected for the study. Ten milliliter of blood sample was collected from each animal using jugular venipuncture. Serum I and plasma was harvested within 2 hours after venipuncture and serum II after 12 hours, and all samples were analyzed for progesterone concentration using the ELISA procedure provided with the kit. The result showed that 88.9% (*n* = 16) of non-pregnant cows had progesterone concentration below 1 ng/ml with mean (±SE) of 0.48 ± 0.75 ng/ml while all pregnant cows had mean (±SE) concentration of 19.3 ± 0.68 ng/ml with individual values ranging from 5.2–38 ng/ml. Progesterone concentration between breeds and sample type did not show statistically significant difference for pregnant and non-pregnant cows. Nonetheless, the results of the experiments are very promising as far as pregnancy diagnosis is concerned in dairy cows from an economic perspective and accuracy; the experiments have to be performed on larger scale to proof repeatability and sensitivity

## 1. Introduction

Developing countries have nearly two third of the world livestock population. However, they produce less than a third of the world's meat and a fifth of its milk. Ethiopia, one of the developing countries in sub-Saharan Africa, has the largest cattle population in Africa with an estimation of 70.2 million heads [1]. The huge cattle resource plays an important role in terms of food security, cash income, capital assets, and social livelihood. However, the country's per capita milk consumption is estimated to be about 20 kg which is far below the average per capital conception of Africa, 40 kg per year [2]. Milk production often does not satisfy the demand due to factors such as poor genetic potential and reproductive performance, malnutrition, traditional management system, high incidence of diseases causing livestock mortality, and socioeconomic factors [3].

Pregnancy diagnosis is an important requirement for successful dairying and to increase the wealth of farmers. It is important to make the right pregnancy diagnosis as soon as possible after insemination so that non-pregnant animals can be observed more closely for heat. Early identification of non-pregnant dairy cows and heifers post-breeding can improve reproductive efficiency and pregnancy rate by decreasing the interval between inseminations and increasing artificial insemination (AI) service rate. Thus, new technologies to identify non-pregnant dairy cows and heifers early after AI may play a key role in management strategies to improve reproductive efficiency and hence profitability of dairy farms. Reproductive management is a major factor affecting profitability in the dairy industry [4]. The ideal pregnancy test should have high sensitivity, correctly identify pregnant animals, high specificity, correctly identify non-pregnant animals, should be simple and inexpensive to conduct under field conditions. There are no simple laboratory tests available to diagnose pregnancy in cattle. Rectal palpation is the oldest and most widely used method for early pregnancy diagnosis in dairy cattle and it has its own disadvantages of rectal bleeding, early embryonic mortality, and extreme stress on the pregnant animal and needs a skilled veterinarian [5]. Advanced methods currently available for pregnancy diagnose such as ultrasonography and radio immunoassay are developed and most used in developed countries and cannot be easily implemented in the rural areas of developing countries [6] such as Ethiopia as they are sophisticated and costly. The most widely used, effective, and reliable method of non-pregnancy detection is determination of progesterone in plasma or milk [7]. Progesterone detection for pregnancy diagnosis in farm animals is an important tool for early pregnancy diagnosis and infertility monitoring with high accuracy which contributes to increase economic efficiency of a farm [4]. Detection of progesterone concentration as a diagnostic tool has been made possible by the introduction of radioimmunoassay techniques [6] and enzyme-linked immunosorbent assay (ELISA) techniques [5]. This quantification could help in pregnancy examination as early after natural or artificial insemination [8], and almost 95% of cows with low progesterone will not be pregnant 24 days after AI [9]. Unfortunately, radioimmunoassay technique is expensive and requires the use of specialized radioisotope facilities and is therefore unsuitable for on-farm use [6].

Progesterone detection methods are basically the same in principle both in animals and human beings. There are mainly five chemical test kits available for detecting the hormone in milk and one for the blood serum in heifers [4,10] In Ethiopia, a pregnancy test kit is not easily available on the market for animals. Even when available, it is twice as costly as the human kit and cannot be easily procured by both researchers and dairy practitioners. On the other hand, human progesterone assay kits are both available on market and less expensive compared to veterinary kits. To alleviate the problems of high cost of diagnostic kits, it is important to develop an alternative simple, reliable, and cheaper test to confirm pregnancy in dairy cattle. This research was initiated with hypotheses: the human ELISA progesterone assay kit would produce the same result in dairy cows. Therefore, the objective of this study was to validate the use of the human progesterone ELISA kit for cattle.

## 2. Materials and Methods

### 2.1. Study Area

The study was conducted at the Holeta Agriculture Research Center (HARC), located 33 km west of Addis Ababa, in West Shoa Zone of Oromia regional state, Ethiopia. It has an altitude of 2400 meter above sea level. The Holeta area has a bimodal rainfall with an average annual rainfall of 1014 millimeter and characterized by cool subtropical weather with minimum and maximum temperature of 6.16°C and 22.3°C, respectively, with a mean relative humidity of 59% [11].

### 2.2. Study Animals and Their Management

The study cows were apparently healthy 22 pregnant (PG) (11 pure Boran and 11 Boran X Holstein–Friesian crosses) and 18 non-pregnant (NPG) (9 Boran and 9 crossbred) owned by HARC. The age of the cows range between 4–10 years and their body condition score was in the range of 4 (medium) to 8 (fat) for both cow groups. All cows were allowed to graze daily for about eight hours and were supplemented with native hay and commercial concentrate. Water was given *ad libitum* twice a day. Lactating cows were machine-milked twice a day. The pregnancy status was determined through rectal palpation 60 days post-breeding. Samples from the pregnant cows were collected after pregnancy was definitively confirmed for cows that were 60 days and more.

### 2.3. Study Design

A total of 20 Boran (11 PG and 9 NPG) and 20 crossbred (11 PG and 9 NPG) cows were selected and used for this study. The PG cows were confirmed 60 days of pregnancy while the NPG ones comprised of cows at least 14 days in postpartum.

Approximately, ten ml of blood sample was collected from all study animals using jugular venipuncture in two plain and one heparinized vacutainer tubes. Serum (Serum I) and plasma were extracted from one plain and one heparinized tubes 2 hours after collection, while another serum (Serum II) was collected from the remaining plain vacutainer after keeping the sample overnight (12 hours) at room temperature to check its possibility whether the serum was affected by overnight storage or not to be used for field condition. Extracted serum and plasma samples were placed in cryovials and stored at −20°C until assay.

The concentrations of progesterone in the collected sample was determined using a competitive ELISA technique (Human^®^, progesterone assay kit, Germany) according to the procedure provided with the kit. The kit has an analytic sensitivity of 0.03 ng/ml. The absorbance of calibrators and specimen was determined using automated ELISA reader system (HUMAREADER, Germany) using a reference wavelength of 630 nm. The concentration of progesterone in collected specimen was interpolated from a dose response curve generated by utilizing serum calibration of known progesterone concentration level in the kit. The kit is based on competitive interaction of progesterone and the hormone-enzyme conjugate for a limited number of immobilized anti-progesterone antibodies and hence the amount of bound hormone-enzyme conjugate is inversely proportional to the concentration of progesterone in the specimen. The concentration range covered by the calibrators is 0 to 40 ng/ml.

### 2.4. Statistical Analysis

The data collected from the experiment were entered into the database management software in Microsoft Excel 2010. The data were analyzed using the general linear model (GLM) procedure of the statistical analysis system (SAS Institute, Kary, NC. USA) to test the fixed effect of breed, pregnancy status, and blood sample type on progesterone concentration. For all cases, *P*-values less than 0.05 were considered statistically significant.

## 3. Results

The study revealed that the observed blood progesterone concentrations in each of the studied animals corresponds to the pregnancy status of the individual animal, which means that all the values were either above 3 ng/ml for PG cows or below 3 ng/ml for NPG ones. The results showed that all of NPG cows had progesterone concentration ≤1.2 ng/ml and all PG cows had progesterone concentration ≥5.2 ng/ml in all samples (Table 1).

In the current study, the mean blood progesterone level was 0.48 ± 0.75 ng/ml (*n* = 18) with values ranging from 0.1 to 1.2 ng/ml for those postpartum NPG cows. Sixteen (88.89%) out of 18 early postpartum NPG cows had a mean progesterone level below 1 ng/ml. The mean (±SE) progesterone level in PG group was 19.31 ± 0.68 ng/ml with individual values ranging from 5.2–38 ng/ml. The blood progesterone concentration between breeds did not show a statistically significant difference within PG and NPG cows (Table2). The progesterone analysis result also indicated that parity number had no statistically significant influence on the progesterone concentration level. The mean progesterone concentration of PG and NPG cows was 23.14 ± 1.18 and 0.56 ± 1.31 ng/ml for plasma, 19.22 ± 1.18 and 0.46 ± 1.31 for serum I and 15.57 ± 1.18 and 0.43 ± 1.31 for serum II, respectively (Table 3).

Numerically, a variation in the progesterone concentration level was seen between plasma, serum I, and serum-II but statistically no significance difference was observed between them. While the progesterone concentrations of serum I, serum II, and plasma were significantly (*P* < 0.001) different between PG and NPG cows.

The summary of progesterone concentration in the different samples both from the PG and NPG group of both breed animals is given in Table 2.

## 4. Discussion

The progesterone concentration level plays an important role in evaluating the reproduction status of the cow. It is an important reproductive hormone to maintain pregnancy and is produced by the corpus luteum of the ovary. Upon measuring, its blood level concentration increases after ovulation and during pregnancy and reduces during follicular growth after corpus luteum regress and parturition [12]. Progesterone concentration is maintained by corpus luteum at a high level in pregnant cows compared with non-pregnant cows [5]. Hence, it is an ideal biomarker whose quantification could be helpful to determine whether the cow is cycling or is pregnant [8]. The human progesterone ELISA kit was able to make the difference between the PG and NPG cattle; in that, the concentration of progesterone in NPG cows was less than 1.2 ng/ml, while it was above 3 ng/ml for the PG cows. Different experiments for veterinary progesterone kits assert similar distinctions between PG and NPG animals [12].

The kit has an analytic sensitivity of 0.03 ng/ml from the human blood sample and can detect the same in animal. The smallest progesterone value in the NPG (0.1) and PG (5.2 ng/ml) found in the present study is closely similar to other findings performed by veterinary kits [12]. The finding of progesterone concentration greater than 3 ng/ml in all samples of PG cattle is strong evidence of the presence of an active corpus luteum in all animals [13, 14]. Similarly, mean progesterone levels in both samples (serum I, serum II, and plasma) collected from NPG cows were lower than 1 ng/ml (*n* = 16) which is an indication of that the animals that were not pregnant and may be in follicular phase of the estrous cycle. Progesterone concentration of serum samples collected from PG and NPG cattle was above 5 ng/ml and below 1 ng/ml, respectively, which indicate that there was no difference in progesterone concentration level if serum samples were extracted immediately or stored overnight for field condition. The cows (*n* = 2) with the highest progesterone (1.2 ng/ml) level in the postpartum group may have had an early return to ovarian activity. This result coincides with the finding of Purohit [14] who measured progesterone and reported that high progesterone concentration indicates that probably the animal is pregnant and low progesterone blood level concentrations at 18 to 24 days of post-breeding can predict the animal is non-pregnant and in follicular phase. The progesterone concentration analysis result indicated that no significant difference was observed between breed and parity in all sample.

In the present study, the progesterone detected in all samples (serum I, serum II, and plasma) collected from PG cows was greater than 3 ng/ml. This result agrees with other reports of validation of the human kit for *Bos taurus*, *Bos indicus,* and crossbred (*Bos taurus* x *Bos indicus*) cows in Cameroon [12]. This result also agrees with the finding of Cavestany et al. [13]. There was also no significant difference in the concentration of progesterone in all samples for both the PG and NPG. It is an indication for concentration of progesterone in serum and plasma samples parallel to the growth and regression of corpus luteum.

## 5. Conclusion

The human progesterone ELISA kit validated in the current study showed similar results to veterinary kits performed at different levels for dairy animals. The smallest and highest values detected in the samples tested were within the ranges of reports for the similar studies in animals. Given the high cost and unavailability of veterinary kits, human progesterone kits offer the best alternative giving equivalent results.

## Figures and Tables

**Table 1:** Mean progesterone concentration in the plasma and serum of pregnant and non-pregnant Boran and crossbred cows in HARC.

		Progesterone concentration (ng/ml)		
		No.	(LSM ± SE)	Range
Pregnancy status	Pregnant	22	19.3 ± 0.68	5.2–38
	Non-pregnant	18	0.48 ± 0.75	0.1–1.2
	*P*-value		*P* < 0.001	

Sample type	Plasma	40	13.0 ± 1.75	11.6–38
	Serum-I	40	10.8 ± 1.75	9.6–37.5
	Serum-II	40	8.8 ± 1.75	5.2–30.25
	*P*-value		*P*=0.238	

Breed	Boran	20	10.6 ± 1.4	12.7–34.1
	Cross	20	11.1 ± 1.4	11.6–38
	*P*-value		*P*=0.805	

LSM = least square mean; SE = standard error of the mean; No = Number of observation.

**Table 2:** Progesterone concentration levels in the different samples drawn from PG and NPG cows irrespective of the breed.

Breed	No.	Samples tested for progesterone concentration (ng/ml)		
		Plasma (LSM ± SE)	Serum I (LSM ± SE)	Serum II (LSM ± SE)
NP Boran	9	0.6 ± 1.89	0.51 ± 1.18	0.49 ± 1.89
NP crossbred	9	0.37 ± 1.89	0.29 ± 1.89	0.27 ± 1.89
PG Boran	11	23.22 ± 1.70	18.95 ± 1.70	13.97 ± 1.70
PG crossbred	11	23.06 ± 1.70	19.49 ± 1.70	17.17 ± 1.70

LSM = least square mean; SE = standard error of the mean. No = number of observations; NPG = non-pregnant; PG = pregnant.

**Table 3:** Overall result of progesterone concentration in the different samples and breeds of cows.

		No	Progesterone concentration in PG (ng/ml)		Progesterone concentration in NPG (ng/ml)	
			(LSM ± SE)	Range	(LSM ± SE)	Range
Sample type	Plasma	40	23.14 ± 1.18	11.6–38	0.56 ± 1.31	0.1–1.2
	Serum-I	40	19.22 ± 1.18	9.6–37.5	0.46 ± 1.31	0.1–1.0
	Serum-II	40	15.57 ± 1.18	5.2–30.25	0.43 ± 1.31	0.1–1.0

Breed	Boran	20	18.71 ± 0.98	12.7–34.1	0.53 ± 1.09	0.1–1.2
	Crossbred	20	19.90 ± 0.98	11.6–38	0.30 ± 1.09	0.1–1.0

LSM = least square mean; SE = standard error of the mean; No = Number of observation; PG = pregnant; NPG=Non-regnant.

## Data Availability

The data used to support the findings of this study are included within the article.
